# Pediatric Soft Tissue and Bone Sarcomas in Tanzania: Epidemiology and Clinical Features

**DOI:** 10.1200/JGO.18.00258

**Published:** 2019-03-27

**Authors:** E. Mithe Siwillis, Nazima J. Dharse, Trish Scanlan, Mamsau Ngoma, Zephania Saitabau Abraham, Josephine W.N. Kahiu, Lynn Million

**Affiliations:** ^1^ Muhimbili University of Health and Allied Sciences, Dar es Salaam, Tanzania; ^2^ University of Dodoma, Dodoma, Tanzania; ^3^ University College London, London, United Kingdom; ^4^ Stanford University School of Medicine, Stanford, CA

## Abstract

**PURPOSE:**

Pediatric sarcomas represent an important group of childhood tumors that require treatment at Muhimbili National Hospital (MNH), the largest pediatric oncology center in Tanzania. Treatment is often adapted from established childhood protocols validated in clinical trials from the United States and the United Kingdom. There are no studies describing the types of pediatric sarcomas most commonly seen in Tanzania to understand similarities and disparities with other countries and which sarcomas to prioritize in adapting treatment protocols. The objective of this study was to establish a baseline of the epidemiologic and clinical features of pediatric sarcomas diagnosed at MNH.

**METHODS:**

Information was collected on clinical and tumor features of all children seen at MNH pediatric oncology unit between 2011 and 2016 with a confirmed histologic diagnosis of either bone or soft tissue sarcoma (STS).

**RESULTS:**

A total of 135 cases were analyzed; 89 (66%) were STS and 46 (34%) were bone sarcomas. There was a slight female predominance (n = 69; 51%), and the mean age (SD) of patients was 6.3 (5.1) years. Greater than 90% (n = 123) of the cases presented with a painless swelling. The commonest STS, accounting for almost three-fourths of the cases (n = 66) was rhabdomyosarcoma (RMS), with embryonal subtype being the most common RMS (n = 49; 74%). Osteosarcoma was the most common bone sarcoma, accounting for greater than 80% (n = 40) of the cases. Ewing sarcoma accounted for less than 15% (n = 6). Most of the patients presented with stage IV disease (n = 57; 87%) and lung was the commonest metastatic site.

**CONCLUSION:**

To our knowledge, this report is the first study documenting the epidemiologic and clinical features of pediatric sarcomas in a modern Tanzanian pediatric hospital. Embryonal RMS and osteosarcomas should be prioritized for adapting treatment protocols from other countries.

**CONTEXT**

**Key Objective** There is a dearth of data regarding pediatric soft tissue and bone sarcomas in Tanzania. To improve outcomes, it is imperative to understand the types of sarcomas diagnosed and treated in Tanzania so treatment protocols from Europe and America can be applied to these rare tumors and modified appropriately to our setting.
**Knowledge Generated** Pediatric soft tissue and bone sarcoma patients in Tanzania present with similar epidemiologic and clinical features as in other developing and developed countries. However, the most notable key difference is that most patients in this setting present with advanced-stage disease.
**Relevance** In view of the relative rarity of these neoplasms, the painless nature of the initial lesion, and hence the late presentation to hospital, awareness within both the community and the medical fraternity should be increased so early detection and treatment are instituted.

## INTRODUCTION

Sarcomas are malignant tumors that arise from mesenchymal tissue at any body site. They form one of the principal groups of rare cancers in Europe, as defined in the RARECARE project.^1^ They can generally be grouped into two main categories: soft tissue and bone sarcomas.^2^

Soft tissue sarcomas (STSs) are rare childhood tumors and make up approximately 12% of pediatric cancers in Europe.^3^ They are further subclassified according to the presumptive tissue of origin and roughly divided into two distinct groups—rhabdomyosarcoma (RMS) and nonrhabdomyosarcoma soft tissue sarcoma (NRSTS)—in which the known chemosensitivity plays an integral role in the management of RMS and is of variable benefit in many NRSTS. Surgery and radiation therapy are routinely used in all STS cases.^4^

Primary bone tumors are rarer and make up approximately 6% of pediatric cancer cases diagnosed annually in Europe.^3^ According to the International Classification of Childhood Cancer classification system, bone cancers are categorized as osteosarcoma (OS), Ewing sarcoma (ES), chondrosarcoma, other specified malignant bone tumors, and unspecified malignant bone tumors.^5,6^ OS and ES make up most of these cases in children.^7^ These bone tumors are broadly classified according to their cytologic features into those that produce osteoid and those that do not. Chemotherapy plays an important role in the management of OS and ES, with surgical resection of the primary tumor necessary for curative treatment in OS, whereas either surgery, radiation therapy or both are commonly considered for treatment of the primary site in ES.^8^

In Tanzania, pediatric sarcomas represent an important group of childhood tumors at Muhimbili National Hospital (MNH), the largest pediatric oncology center in the country. Management is guided through treatment protocols adapted from clinical trials run by Children’s Oncology Group and the International Society of Pediatric Oncology, with therapy modified on the basis of drug availability and surgical and radiation therapy resources. Currently, there are no baseline data on the frequency and types of pediatric sarcomas in Tanzania. The objective of this study was to establish the epidemiology and clinical features of children with bone sarcomas and STSs to better focus and prioritize treatment protocols and resources toward the more common pediatric sarcomas in Tanzania.

## METHODS

### Study Setting and Data Collection

This was a descriptive retrospective review of pediatric patients aged between 0 to 18 years with a histopathologically confirmed diagnosis of either an STS or bone sarcoma at MNH Pediatric Oncology Unit from January 2011 to December 2016. Data extraction forms were used to retrieve data from patients’ records, which were stored in manual files and or on the computer, and these captured demographic and clinicopathologic characteristics. The statistical analysis was performed using SPSS, version 20.0 for Windows (SPSS, Chicago, IL). Continuous variables were summarized by reporting means, medians, standard deviations, and range. Categorical variables were summarized by reporting frequencies and percentages, and using bar graphs for general description.

### Ethical Consideration and Consent Process

Ethical clearance was obtained from the Muhimbili University of Health and allied sciences Research Ethics and Publication Committee before implementation of this study. A waiver of informed consent was also requested and granted by the same committee, and permission to conduct the study was obtained from MNH, per hospital management protocols.

## RESULTS 

A total of 135 cases of histologically confirmed bone sarcoma or STS fulfilled the criteria and were included in the final analysis; 89 (66%) of the cases were STS and 46 (34%) were bone sarcomas. Table 1 lists clinical and tumor characteristics of all eligible cases. There was a slight overall female predominance of 69 patients (51%). The mean age for all patients was 6.3 years; most STS patients (n = 37; 42%) were younger than 5 years of age, and most patients with bone sarcomas (n = 21; 46%) were between 10 to 15 years of age.

**TABLE 1:**
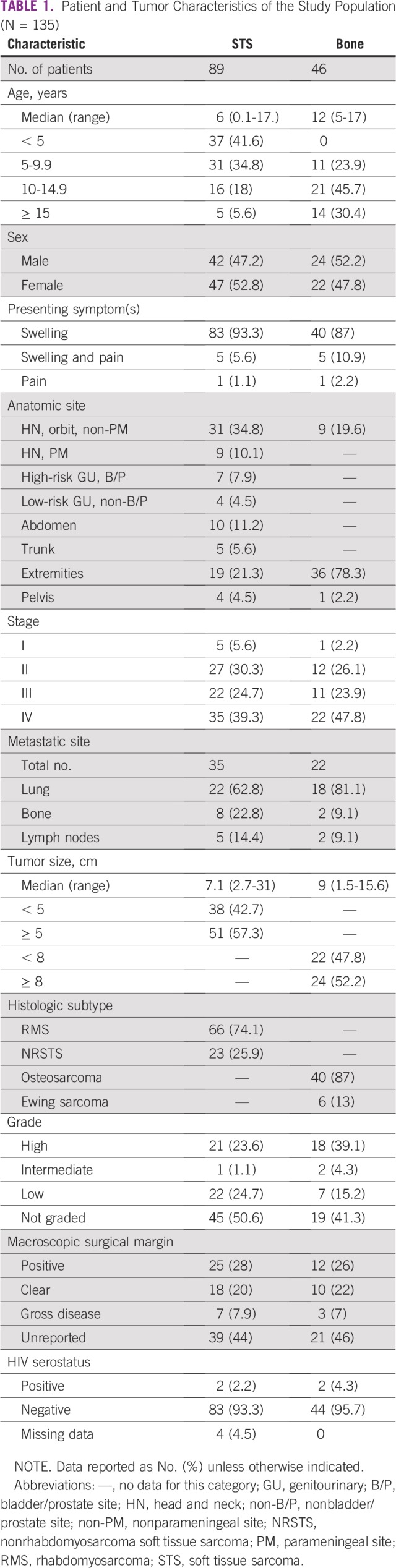
Patient and Tumor Characteristics of the Study Population (N = 135)

Most patients presented with a painless swelling. The commonest site for the STS category was the head and neck; the extremities were the more common sites for the bone sarcomas. Most patients presented with stage IV disease, with lung being the most common site of distant metastasis. Most patients presented with large tumors (> 5 cm for the STS and > 8 cm for the bone sarcomas). RMS accounted for almost 66 (75%) of the patients with STS and OS accounted for 40 (87%) of patients with bone sarcomas. Most of the patients did not have histologic grade or macroscopic surgical margin reported and there was no documentation of surgical margin status on the pathologic report. There was no HIV correlation to these malignancies.

**FIG 1:**
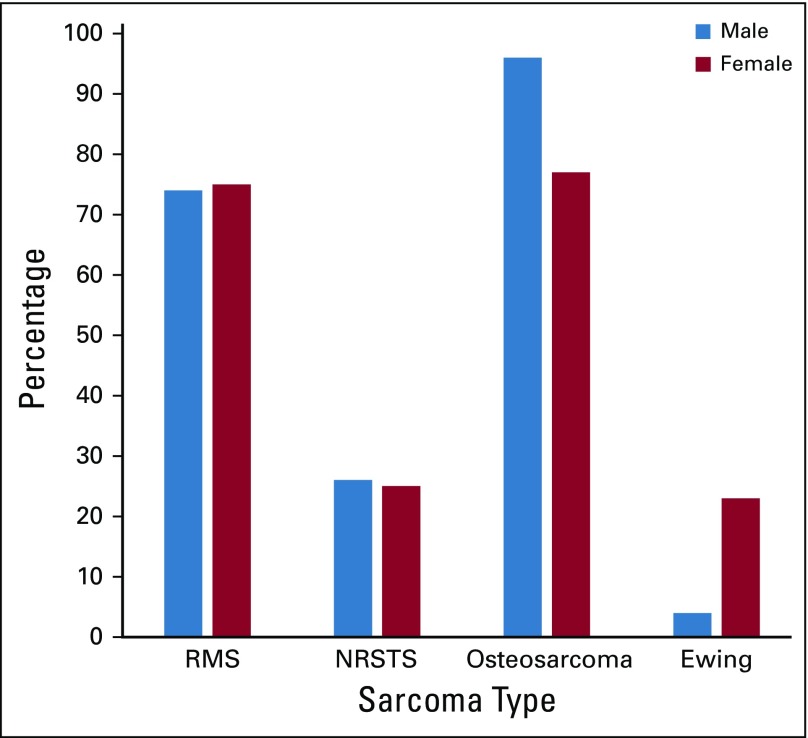
Distribution of soft tissue and bone sarcomas by sex in relation to the specific histologic subtypes. RMS, rhabdomyosarcoma; NRSTS, nonrhabdomyosarcoma soft tissue sarcoma.

Figure 1 depicts the frequency of pediatric sarcomas based on histology in relation to sex and shows OS was more common in males than in females (96% *v* 78%), whereas ES was more common in females (22%) compared with males (4%). RMS and NRSTS had a similar incidence in both sexes.

Table 2 shows that embryonal RMS and alveolar RMS occurred more commonly in the head and neck region. The second most common site for embryonal RMS was the high-risk and low-risk genitourinary sites collectively (n = 11; 16%) and the third most common site was equally distributed between the extremities and abdomen (n = 6; 12%), whereas the second most common site of alveolar RMS was in an extremity. NRSTS most commonly involved the extremities (n = 9; 39%). OS and ES were commonest in the extremities (n = 32; 80% and n = 4; 67%), respectively.

**TABLE 2:**
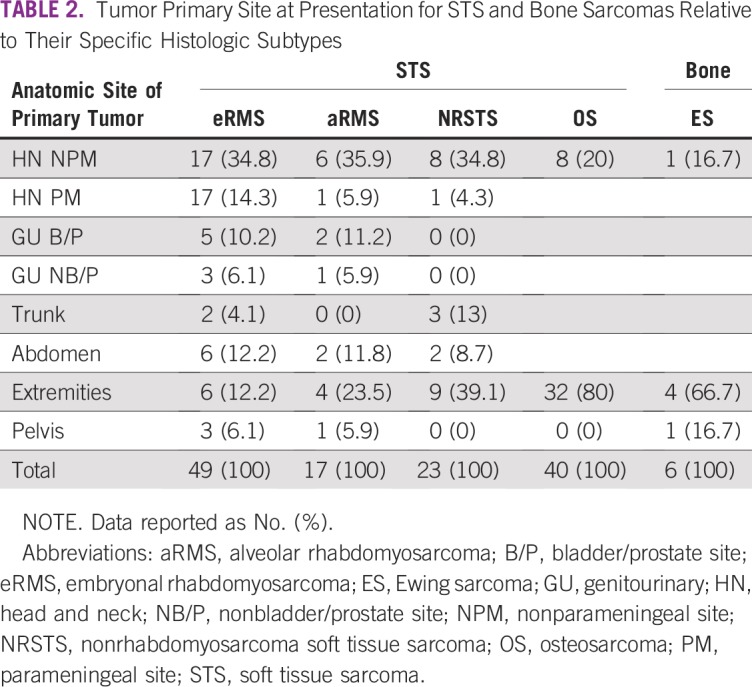
Tumor Primary Site at Presentation for STS and Bone Sarcomas Relative to Their Specific Histologic Subtypes

Table 3 shows that most patients with RMS presented with advanced-stage disease—stage IV disease (n = 27; 41%) and stage III disease (n = 18; 27%); those with NRSTS presented more commonly with earlier stage disease—stage II (n = 10; 44%); however more than one-third of the NRSTS cases presented with metastatic disease—stage IV (n = 8; 35%). Most patients with OS and ES presented with stage IV disease (n = 18; 45% and n = 4; 67%), respectively.

**TABLE 3:**
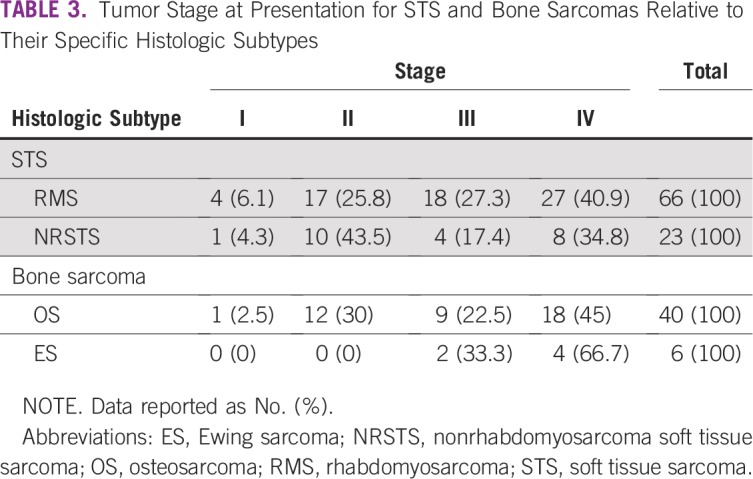
Tumor Stage at Presentation for STS and Bone Sarcomas Relative to Their Specific Histologic Subtypes

**FIG 2:**
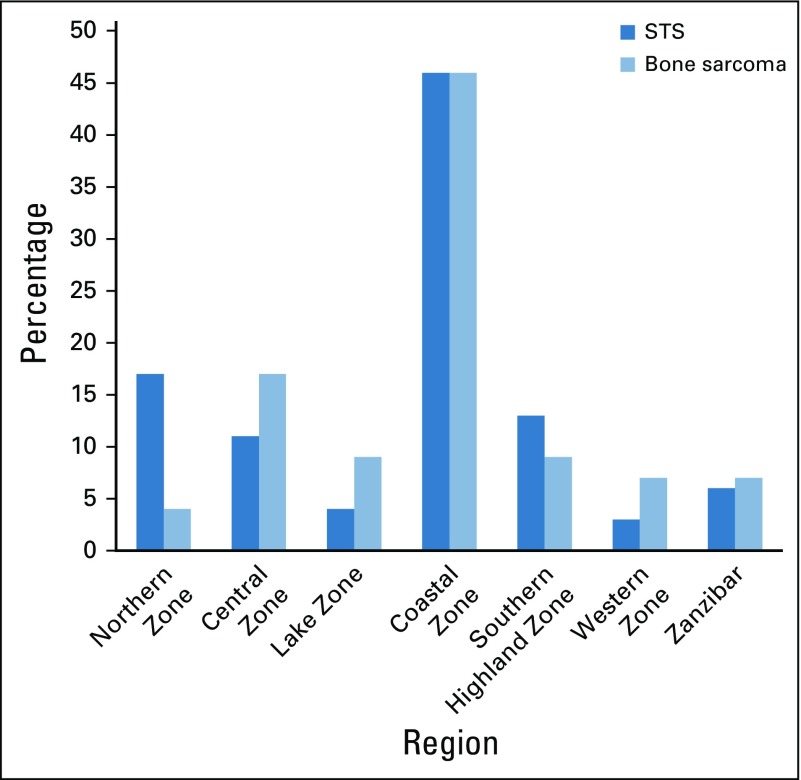
General distribution of soft tissue sarcoma (STS) and bone sarcomas by geographic regions in Tanzania.

Most of the patients came from the Coastal (ie, Dar es Salaam, Tanga, and Morogoro), the Central (ie, Dodoma and Singida) and the Northern (ie, Arusha and Kilimanjaro) zones. The Western and Lake (ie, Tabora, Kigoma, Shinyanga, Kagera, Mwanza, and Mara) zones had the fewest cases (Fig 2).

## DISCUSSION

To our knowledge, this is the first study to document the epidemiology and clinical features of pediatric bone sarcomas and STSs in Tanzania and provides important baseline data with which to compare results with that of other countries and an opportunity to prioritize treatment protocol development and modification.

A key finding in this study is that similar to other countries, embryonal RMS accounts for the overwhelming majority of all sarcomas seen at MNH.^9-11^ There are few epidemiologic studies from other African countries, but reports from Nigeria^10^ and South Africa^11^ also show RMS to be the most common STS, with the embryonal RMS variant being the most common histologic subtype.^11^

Embryonal RMS is one of the most curable of the sarcomas. Clinical trials standardizing management for specific anatomic sites can be adopted and modified to the pediatric oncology unit at MNH and so offer promise for potentially curative treatment, depending on availability of resources such as chemotherapy drugs, surgery, and radiation therapy.

Two other key findings that differ from reports of pediatric sarcomas in other countries are that very few cases of ES were diagnosed at MNH during the 6-year study period. This is in keeping with the racial disparity reported in the SEER series,^5,6^ in which ES is noted to have an approximately six-fold higher incidence in white ethnic children as compared with children of black ethnicity.^5,6^ Thus, modifying treatment protocols for ES would not be a high priority in our setting.

Most importantly, a disparity noted for all pediatric sarcomas in Tanzania is the overwhelming majority (n = 57; 42%) of patients presenting with advanced-stage disease. The importance of this finding is that curative therapy is not possible when children present with distant metastatic disease, and symptom control may be the best therapy that can be offered. Van Der Schyff and Stefan^11^ in South Africa also reported similar results, with greater than 40% incidence of distant metastasis at diagnosis, with bone sarcoma accounting for the highest frequency of those presenting with distant metastasis. In comparison, a report from India^12^ showed that only 15% to 25% of newly diagnosed RMS present with distant metastasis. However, when found, the lung is also the most common metastatic site (60%), followed by bone marrow (30% to 40%), bone (10%), and lymph nodes (5%), depending on the site of the primary tumor.

Other key disparities noted in our study include the similar incidence of males and females at presentation, whereas in the SEER series of 1975 to 1995, males had a slightly higher incidence than females.^5,6^ A male predominance (58%) in RMS cases compared with females (42%) was also noted in a South African study by Van Der Schyff and Stefan.^11^ Our study also slightly differs in the age distribution for alveolar RMS, which was more commonly seen in younger patients (< 5 years of age), whereas in the SEER series, it is more common for patients to present at an older age.^5,6^

Similarities include clinical presentation of a painless swelling and anatomic site of presentation including head and neck for RMS, whereas the extremities were more common for NRSTS and bone sarcomas. Our findings are in keeping with studies from the United States,^5^ Nigeria,^10^ and India.^13^ The SEER analysis^5,6^ shows that the most frequent site of bone sarcoma development was the metaphysis of the long bones of the lower limbs for OS and the central axis (ie, pelvis and axial skeleton) for ES. In a study by Ferreira et al,^14^ the metaphysis of long bones of the lower limbs was the most common site for OS: the distal femur (58%) followed by the proximal tibia (42%).

There are several limitations in this study, including that is a retrospective review over a relatively short time and the data were obtained from one hospital, which may not be representative of the entire country. Future directions include reviewing treatment and outcome for pediatric sarcomas treated at MNH to compare treatment results with other countries.

To our knowledge, this is the first study to document the epidemiologic and clinical features of pediatric STS and bone sarcomas in Tanzania. We demonstrate that although there are many similar findings compared with other countries, including embryonal RMS being the most common pediatric sarcoma where treatment protocols are available for adaption, the most notable differences from other countries are that most patients in our study presented with advanced-stage disease, which reduces the chances of curative treatment, and recognizing a limited need to adapt ES treatment protocols in our setting.^13^

## References

[1] Stiller CA Trama A Serraino D et al Descriptive epidemiology of sarcomas in Europe: Report from the RARECARE project Eur J Cancer 49 684 695 2013 23079473 10.1016/j.ejca.2012.09.011

[2] Skubitz KM D’Adamo DR Sarcoma Mayo Clin Proc 82 1409 1432 2007 17976362 10.4065/82.11.1409

[3] Sultan I Qaddoumi I Yaser S et al Comparing adult and pediatric rhabdomyosarcoma in the surveillance, epidemiology and end results program, 1973 to 2005: an analysis of 2,600 patients J Clin Oncol 27 3391 3397 2009 19398574 10.1200/JCO.2008.19.7483

[4] ESMO/European Sarcoma Network Working Group Soft tissue and visceral sarcomas: ESMO clinical practice guidelines for diagnosis, treatment and follow-up Ann Oncol 23 102 112 2012 (suppl 7) 10.1093/annonc/mds253 22997462

[5] Ries LAG Smith MA Gurney JG et al (eds): Cancer Incidence and Survival Among Children and Adolescents: United States SEER Program 1975-1995 NIH Pub No 99-4649 Bethesda, MD National Institutes of Health 1999 p 179

[6] Hayat MJ, Howlader N, Reichman ME, et al: Cancer statistics, trends, and multiple primary cancer analyses from the Surveillance, Epidemiology, and End Results (SEER) Program. Oncologist. 12(1):20–37 2007 10.1634/theoncologist.12-1-20 17227898

[7] Kaste SC Imaging pediatric bone sarcomas Radiol Clin North Am 49 749 vii 2011 21807172 10.1016/j.rcl.2011.05.006 PMC4725719

[8] Hogendoorn PCW Athanasou N Bielack S et al Bone sarcomas: ESMO Clinical Practice Guidelines for diagnosis, treatment and follow-up Ann Oncol 21 204 213 2010 (suppl 5) 20555083 10.1093/annonc/mdq223

[9] Kachanov DY Dobrenkov K V Abdullaev RT et al Incidence and survival of pediatric soft tissue sarcomas in Moscow region, Russian Federation, 2000-2009 Sarcoma 10.1155/2012/350806 PMC3337554 22566750

[10] Brown BJ Owuwasola AO Childhood rhabdomyosarcoma in Ibadan, Nigeria: 1984-2003 Ann Trop Paediatr 26 349 355 2006 17132301 10.1179/146532806X152881

[11] Van Der Schyff A Stefan DC Clinical characteristics and outcome of rhabdomyosarcoma in South African children. African J Haematol Oncol 2010 http://hdl.handle.net/10019.1/38598

[12] Kapoor G Das K Soft tissue sarcomas in children Indian J Pediatr 79 936 942 2012 21935710 10.1007/s12098-011-0560-4

[13] Ramaswamy A Rekhi B Bakhshi S et al Indian data on bone and soft tissue sarcomas: A summary of published study results South Asian J Cancer 5 138 145 2016 http://www.ncbi.nlm.nih.gov/pmc/articles/PMC4991135/27606300 10.4103/2278-330X.187587 PMC4991135

[14] Ferreira N Marais L. C. Osteosarcoma presentation stages at a tumour unit in South Africa S Afr Med J 102 673 676 2012 22831944 10.7196/samj.5835

